# Mental Health Awareness: Stigma and Help-Seeking Among Portuguese College Students

**DOI:** 10.3390/healthcare12242505

**Published:** 2024-12-11

**Authors:** Paula Saraiva Carvalho, Nádia Pombal, Jorge Gama, Manuel Loureiro

**Affiliations:** 1 Department of Psychology and Education, University of Beira Interior, 6200-209 Covilhã, Portugal; nadia.pombal@ubi.pt (N.P.); loureiro@ubi.pt (M.L.); 2 Research Center in Sports Sciences, Health Sciences and Human Development (CIDESD), 5000-801 Vila Real, Portugal; 3 Centre of Mathematics and Applications, University of Beira Interior, 6201-001 Covilhã, Portugal; jgama@ubi.pt

**Keywords:** mental illness stigma, help-seeking behaviour, college students, mental health, cluster analysis

## Abstract

Background/Objectives: Help-seeking—that is, the attempt to attain external help for mental health, be it from formal or informal sources—can be described as an adaptive coping process. Mental illness stigma is the most frequently identified barrier that prevents students from seeking psychological help. This study analyzed college students’ beliefs about mental illness and attitudes toward formal psychological help-seeking. Methods: Two hundred and eighty-two students from the first and third undergraduate years of Psychology, Sociology, Fashion Design, and Sports Science courses participated. The majority of the sample (75.4%) was female, while only 24.6% was male, with a mean age of 20.04 years. The scales used were the Inventory of Beliefs about Mental Illness (IBMI), the Inventory of Attitudes Toward Seeking Mental Health Services (IATSMHS), and a sociodemographic questionnaire. Results: The results revealed that females, third-year students, and Psychology students had fewer stigmatizing beliefs about mental illness and more positive attitudes towards help-seeking. Moreover, there were statistically significant differences in beliefs and attitudes in relation to psychological support and familiarity with mental illness. We also glimpsed the impact of the COVID-19 pandemic on students’ mental health, and observed a change in opinions and attitudes toward mental illness during this period. Correlation analysis showed negative correlations between stigmatizing beliefs and attitudes toward seeking help. Finally, a cluster analysis identified two profiles of individuals that reflected different levels of stigma and help-seeking attitudes. Conclusions: This study delineated two distinct groups of students, which is relevant as it allows us to trace profiles to outline more uniform intervention groups and, in turn, implement new and improved interventions that are better adapted to the specific needs of college students.

## 1. Introduction

Stigma was initially defined in 1963 by Erving Goffman as a highly derogatory social attribute, used by a group of people to alienate an individual or group with specific characteristics that separate them from the so-called “normal”, resulting in rejection, discrimination, and social exclusion [1].

Link and Phelan describe stigma as the convergence of four interrelated components. First, a group or individual is labelled according to a human difference relevant to society. The second component involves a label that associates the person with certain undesirable characteristics, resulting in the formation of a stereotype. The third component implies the distinction and separation of groups—“us” from “them”. Finally, the labelled person experiences rejection, discrimination, exclusion, and loss of status [2]. This marginalization happens when the three components are perpetuated by a group with greater political, cultural, or economic power [2,3].

Stigma is defined as public stigma when the general population exercises negative attitudes and beliefs toward people with mental illness, while self-stigma applies when public stigma is internalized by the individual with mental illness, resulting in negative emotional reactions such as low self-esteem. Both of these forms of stigma are generally understood to encompass three cognitive and behavioural dimensions, namely, stereotypes, prejudice, and discrimination [4].

Help-seeking—that is, the attempt to attain external help for mental health, be it from formal or informal sources—can be described as an adaptive coping process. It includes all stages of the process, from the intention to engage in mental healthcare, to the behaviour of seeking help [5].

Mental illness stigma is the most frequently identified barrier that prevents students from seeking psychological help [6,7,8,9], especially with respect to the avoidance of the “mentally ill” label caused by public stigma [4]. Fear of stigma related to mental health problems is high among young people, who often do not want peers to know about their struggles out of shame or fear of being mocked. This fear is also related to concerns about the confidentiality of professional mental health services [6].

The literature recognizes familiarity with mental illness as a variable that moderates the development and expression of stigma. Members of the general public who are familiar with mental illness, whether through education, experience with family members or significant others, or personal experience with mental health problems, show more positive attitudes toward mental illness and less desire for social distancing [10,11,12]. Moreover, Nohr et al. found that being familiar with people with mental illness might reduce label avoidance, which in turn decreases negative help-seeking attitudes [13].

The COVID-19 pandemic has significantly impacted the mental health of college students, leading to a notable increase in depression and anxiety symptoms [14,15,16]. Despite recognizing the need for psychological help, many students often do not seek it. Instead, young people tend to turn to friends or family rather than healthcare professionals [14]. The underutilization of mental health services among college students is not only due to a lack of information, but also due to misinformation about mental health and the fear of being stigmatized [14,15,16].

The first aim of this study was to investigate differences in beliefs about mental illness and help-seeking attitudes based on demographic data. The second aim of the study was to assess the influence of the COVID-19 pandemic on opinions and attitudes toward mental illness. Finally, the third goal of the study was to identify the different profiles of participants.

## 2. Materials and Methods

### 2.1. Measures

#### 2.1.1. Sociodemographic Questionnaire

A sociodemographic questionnaire was built to acquire sociodemographic information, including gender, age, nationality, university year and course, etc. Questions were also included that pertained to familiarity with mental illness (e.g., having a family member/other with mental illness and regularity of contact), whether participants had any type of psychological support, and if they had ever felt the need to seek psychological help during or after the COVID-19 pandemic, and from whom (e.g., psychologist, psychiatrist, family doctor). Finally, the students were asked whether they felt that their opinions and attitudes about mental illness had changed since the pandemic, and they were asked to describe in what way.

#### 2.1.2. Inventory of Beliefs About Mental Illness (IBMI)

This scale measures stigma, and was developed for the Portuguese population by Loureiro et al. [17]. The 45-item version of the IBMI was used in this study, with items rated on a five-point Likert scale (1 = completely disagree; 6 = completely agree).

The scale encompasses the following factors: Incurability (belief that mental illness has a chronic and incurable nature and that medication is necessary to control it; it also refers to a lack of competence in assuming work and family responsibilities); Disease Recognition (a non-stigmatizing view of mental illness, associated with acceptance, confidence in treatment, and rehabilitation); Dangerousness (belief in the mentally ill as dangerous, given the unpredictability of their behaviour); Illness as a Cause of Stigma and Discrimination (personal belief that mental illness can create stigma and discrimination in the patient’s intimate circles); Personal Responsibility (belief that the individual is responsible for their own health/illness); Illness as a Medical Condition (belief that mental illness is a clinical condition that requires medication) [17].

Regarding the scale’s internal consistency, in this study, an alpha of 0.75 was obtained for the global scale, which reflects good internal consistency. Furthermore, a value of 0.67 was obtained for Dangerousness and 0.66 for Incurability, which reflects an acceptable internal consistency; 0.54 was obtained for Disease Recognition and 0.54 for Illness as a Cause of Stigma and Discrimination, indicating a weak internal consistency [18]. Considering the unacceptable values of Illness as a Medical Condition (α = 0.48) and Personal Responsibility (α = 0.41), they were not used in the statistical analysis.

#### 2.1.3. Inventory of Attitudes Toward Seeking Mental Health Services (IATSMHS)

The Portuguese version of this scale, adapted by Fonseca et al. [19], was used to measure help-seeking attitudes. The IATSMHS was originally developed by Mackenzie et al. [20] to analyze attitudes toward seeking formal help, understood as an evaluative reaction to seeking help for dealing with psychological or emotional problems.

The scale consists of 24 items, organized into three dimensions: Psychological Openness, which reflects the degree to which individuals are open to recognizing their psychological problems and open to the possibility of seeking professional help; Propensity to Seek Help, which concerns the extent to which individuals believe they are willing and able to seek help; and Indifference to Stigma, which is related to how much individuals care about what significant others might think if they know they are receiving psychological help [20]. The items are rated on a five-point Likert scale (0 = disagree; 4 = agree), with some items being scored in reverse [19].

Concerning internal consistency, the authors found a Cronbach’s alpha value of 0.83 for the total scale [19]. In this study, the alpha values for the global scale were 0.79 and 0.77 for Indifference to Stigma, which reflects a good internal consistency. As for Propensity to Seek Help, a value of 0.69 was obtained, considered as acceptable, and for Psychological Openness, a value of 0.52 was obtained, considered as weak [18].

### 2.2. Procedure

The study was performed in accordance with the Declaration of Helsinki regarding research on human participants, and was approved by the Ethics Committee of the University of Beira Interior (CE-UBI-Pj-2021-075). Data were collected using questionnaires completed in classroom settings. Before distributing the questionnaires, participants were informed of the purpose of the study and the voluntary and anonymous nature of their participation. The right to accept or decline participation was assured. Participation in the study was not a course requirement. Only the researchers had access to the data and all students signed an informed consent form. The inclusion criteria for the study were as follows: (1) to be above 18 years old, (2) to be attending the 1st or 3rd undergraduate years of Psychology, Sociology, Fashion Design, and Sports Science courses at the University of Beira Interior, and (3) being able to answer the research protocol in Portuguese.

### 2.3. Statistical/Data Analysis

The data were processed using the Statistical Package for the Social Sciences (SPSS), version 29.0.1. Firstly, the descriptive statistics of the sample were analyzed by calculating the mean (M), standard deviation (SD), and minimum and maximum values for the quantitative data, and frequencies and percentages for the categorical data.

The independent samples *t*-Test or analysis of variance (ANOVA) was conducted to compare two or four groups. For the latter, post hoc comparisons were performed using the LSD test (Least Significance Difference) with Sidak’s correction. Although there was a violation of the normality assumption in many cases, verified by the Kolmogorov–Smirnov test with Lilliefors correction, the aforementioned parametric approach was used due to the sample size (at least 54 in the considered groups, with a total sample size equal to 282; therefore, a sample of *N* > 30, according to the Central Limit Theorem). However, it was necessary to resort, in some cases, to the non-parametric Mann–Whitney test, due to the existence of excessively influential outliers. As a precaution, in cases where we used parametric analyses, we also carried out alternative non-parametric analyses, and verified that the conclusions did not change. When applicable, the assumption of homogeneity of variances was verified by Levene’s test. This ANOVA assumption was not violated.

Pearson’s correlation coefficient was used to examine the correlation between the IATSMHS and IBMI factors. Pearson’s chi-square test was performed to verify the association between two categorical variables.

Finally, a cluster analysis was performed using the Two-Step Cluster method [21], which allowed the simultaneous inclusion of both quantitative and categorical variables in the analysis. For this exploratory analysis, we considered 15 variables: 7 categorical (course, gender, family member with mental illness, college year, need for psychological support, psychological support, and change in opinion/attitudes) and 8 quantitative (age, psychological openness, dangerousness, propensity to seek help, illness as a cause of stigma and discrimination, indifference to stigma, incurability, and disease recognition); nothing needed to be specified about the clusters to be retained. This analysis can also be configured to automatically exclude outliers that could change the results. However, with or without outliers, the two clusters found remained unchanged.

All hypothesis tests were considered significant when the respective *p*-value (*p*) did not exceed the 5% significance level.

## 3. Results

### 3.1. Participants’ Characteristics

The participant sample consisted of 282 college students. The majority were female (*N* = 212, 75.4%), and 69 were male (24.6%). They were aged between 18 and 41 years, with a mean age of 20.04 (SD = 2.48). Table 1 provides a more detailed description of the sample.

### 3.2. Statistical Analysis of IBMI and IATSMHS Factors

First, the descriptive statistics of all the subscales were analyzed. Concerning the IBMI scale, the subscale with the highest mean was Disease Recognition (M = 50.42, SD = 4.59), followed by Incurability (M = 28.45, SD = 6.89), Dangerousness (M = 17.02, SD = 5.01), and Illness as a Cause of Stigma and Discrimination (M = 13.70, SD = 4.02). As for the IATSMHS, the highest mean level related to Indifference to Stigma (M = 28.25, SD = 4.53), followed closely by Propensity to Seek Help (M = 25.04, SD = 4.82) and Psychological Openness (M = 22.27, SD = 4.55) (Table S1).

#### 3.2.1. Effect of Gender on IBMI and IATSMHS Factors

The *t*-Test for independent samples or the Mann–Whitney test was performed to verify the differences between the IBMI and IAHTSM factors by gender. Significant differences (*p* < 0.05) were found for all variables. Male students presented higher scores for stigmatizing beliefs, namely Incurability (M = 29.92), Illness as a Cause of Stigma and Discrimination (M = 14.62), and Dangerousness (M = 18.30). Female students presented higher scores for Disease Recognition (M = 50.71). Concerning the IATSMHS subscales, gender had a statistically significant effect on help-seeking attitudes, specifically Psychological Openness (U = 10,235.50; *p* < 0.001), Propensity to Seek Help (U = 9034.50; *p* < 0.001), as well as on the global score (U = 9588.50; *p* < 0.001), with higher scores in female students (Table S2).

#### 3.2.2. Effect of College Year on IBMI and IATSMHS Factors

Statistically significant differences exist in regard to Incurability (t(271) = 3.093; *p* = 0.002) and Dangerousness (t(273) = 3.645; *p* < 0.001), as well as the global score (t(247) = 3.421; *p* < 0.001). In all factors, first-year students displayed the highest scores (M = 29.50; M = 17.91; M = 149.60, respectively). As for the IATSMHS, a statistically significant difference was found between college years on the Psychological Openness subscale (t(278) = −2.325; *p* = 0.021), with a higher mean level in third-year students (M = 23.02) (Table S3).

#### 3.2.3. Effect of Course on IBMI and IATSMHS Factors

The tests revealed statistically significant differences for Incurability (F = 8.459; *p* < 0.001), Dangerousness (F = 10.347; *p* < 0.001), and the global score (F = 5.807; *p* < 0.001). Fashion Design students displayed the highest scores for Incurability (M = 30.58), followed by Sociology (M = 29.63), Sports Science (M = 28.77), and Psychology students (M = 25.59). Sports students showed the highest mean for Dangerousness (M = 18.94), followed by Design (M = 18.10), Sociology (M = 16.55), and Psychology students (M = 14.89). Concerning the global score, Design students presented the highest scores (M = 150.84), followed by Sports (M = 149.54), Sociology (M = 146.50), and Psychology students (M = 141.20) (Table S4). Subsequently, a post hoc test was conducted to determine the differences between the four groups. The mean I–J difference was significant between Psychology and Sociology students concerning Incurability (*p* = 0.04); between Psychology and Design students (*p* < 0.001), as well as Psychology and Sports Science students (*p* < 0.05), on all subscales; and finally, between Sociology and Sports Science students concerning Dangerousness (*p* = 0.046) (Table S5).

Concerning all factors of the IATSMHS, statistically significant differences between courses (*p* < 0.001) were evident. The highest scores were found in Psychology students for Psychological Openness (M = 24.29), Propensity to Seek Help (M = 27.23), Indifference to Stigma (M = 29.88), and the total score (M = 81.35), followed by Sociology, Fashion Design and Sports Science students, in descending order (Table S6). Once again, a post hoc test was performed to analyze the group comparisons. The mean I–J difference was significant between Psychology and Fashion Design students and between Psychology and Sports Science students concerning all dimensions (*p* < 0.05); it was also significant between Sociology and Psychology students (*p* = 0.009) and, finally, Sociology and Sports students (*p* = 0.032) concerning the global score (Table S7).

#### 3.2.4. Effect of Psychological Support on IBMI and IATSMHS Factors

The results confirm the presence of a statistically significant difference concerning the Dangerousness factor (t(271) = −3.315, *p* < 0.001), with a higher score in participants who did not have formal psychological support at the time of asking (M = 17.55) compared to the students who did (M = 15.11). As for IATSMHS dimensions, statistically significant differences were found between groups for Psychological Openness (t(276) = 3.835; *p* < 0.001) and Propensity to Seek Help (t(105.705) = 6.135; *p* < 0.001). The participants with psychological support presented a higher mean level of Psychological Openness (M = 24.27) and Propensity to Seek Help (M = 27.91). Concerning the global score, a significant difference (t(272) = 4.137; *p* < 0.001) was also found, with higher scores in participants with psychological support (M = 80.55) (Table S8).

#### 3.2.5. Effect of Familiarity with Mental Illness on IBMI and IATSMHS Factors

The results obtained showed the presence of a statistically significant difference in Disease Recognition (t(262) = 2.474; *p* = 0.014), with higher scores in participants who reported having a family member diagnosed with a mental disorder (M = 51.46). Concerning the factors of the IATSMHS, the results showed that there was a statistically significant difference in the Propensity to Seek Help factor (t(271) = 2.873; *p* = 0.004), indicating that those who reported having a family member with mental illness were more likely to seek help (M = 26.20) (Table S9).

#### 3.2.6. Correlations Between IBMI and IATSMHS Factors

The strongest correlations were found between Illness as a Cause of Stigma and Discrimination and Indifference to Stigma (r = −0.659; *p* < 0.001), which reflected a moderate and negative correlation, as well as between Illness as a Cause of Stigma and Discrimination and the IATSMHS total score (r = −0.563; *p* < 0.001). Illness as a Cause of Stigma and Discrimination was also negatively correlated with Psychological Openness (r = −0.379).

Incurability was significantly and negatively correlated with Psychological Openness (r = −0.278), Indifference to Stigma (r = −0.286), and total score (r = −0.252). Dangerousness was also significantly and negatively correlated with Psychological Openness (r = −0.389), Indifference to Stigma (r = −0.350), and the IATSMHS total score (r = −0.396).

The IBMI total score was significantly and negatively correlated with Psychological Openness (r = −0.398), Indifference to Stigma (r = −0.391), and the IATSMHS total score (r = −0.364). The remaining significant correlations can be considered null (Table 2).

#### 3.2.7. Statistical Analysis Under the Effect of Clusters Retained by Two-Step Cluster Analysis

Finally, we performed a cluster analysis (Table 3) from which two clusters or profiles of participants were identified. The quality of this analysis was considered acceptable (silhouette measure = 0.2). The profiles are described in the following subsections.

Self-Aware Students—the largest group (*N* = 146, 60.8%), and mainly composed of female students (75.5%), with an average age of 20.50 years. Most of these participants studied Psychology (91.5%) and were in their third year of college (83.0%). The majority reported currently having psychological support (94.1%) and knowing a family member with a mental disorder (89.9%). They also revealed feeling the need to seek psychological help during or after the COVID-19 pandemic (79.6%) and admitted to having changed their attitudes toward people with mental illness during that time (67.1%). This group showed—in order of predictive importance—higher psychological openness (M = 23.88), propensity to seek help (M = 26.49), and indifference to stigma (M = 29.38), when compared to non-self-aware students.

Prejudiced Students—consisted mainly of male students (*N* = 94, 87.5%), with a mean age of 19.31 years. Most of these participants studied Sports Science (87.0%) and were in their first year of university (55.0%). In this group, students reported not currently benefiting from psychological support (48.1%) and not knowing a family member with mental illness (53.8%). They revealed not having felt the need to seek psychological help during the pandemic (55.9%), nor having changed their attitudes and opinions about mental illness (48.5%). This group showed higher levels of stigmatizing beliefs, such as dangerousness (M = 19.42), incurability (M = 30.49), and illness as a cause of stigma and discrimination (M = 15.29), compared to non-prejudiced students.

## 4. Discussion

The present study contributes to scientific research by providing some theoretical insights into mental illness stigma and help-seeking attitudes and their relationship with other variables. Furthermore, it confirms the association between stigma related to mental illness and less active help-seeking for mental health problems, particularly among college students [6,7,22,23,24]. This association occurs as the presence of stigmatizing or negative beliefs about mental illness tends to indicate a greater stigma associated with treatment, leading to negative attitudes related to psychological help-seeking, in the form of lower psychological openness, propensity to seek help, and indifference to stigma.

The results also revealed a “gender effect” perspective on stigma, as recognized by the literature. As such, it was found that male students, compared to females, exhibited more stigmatizing beliefs about mental illness, including incurability, dangerousness, and the belief in illness as a cause of stigma and discrimination. This difference may depend on a different conceptualization of mental illness, as women tend to have psychosocial, rather than biological, explanations for mental illness, the latter being often associated with more negative attitudes [25]. Moreover, women seem to exhibit higher levels of social empathy [26]. Consistent with previous findings, female students were found to be more likely to seek help from professional sources [7,22]. Several studies [23,27] have found that, overall, male college students have unfavourable help-seeking attitudes and weak intentions to seek help for mental health problems. These differences may be related to traditional gender roles, meaning that society expects men to be strong, resilient, and independent, and help-seeking is seen as a sign of weakness. This stalwart attitude, in turn, tends to foster negative attitudes toward their need to seek help for mental health problems [28].

Concerning academic year, on the one hand, first-year students were revealed to have more stigmatizing beliefs about mental illness (e.g., incurability and dangerousness), compared to third-year students. On the other hand, third-year students appeared to be more psychologically open to seeking help. In support of these results, some authors [29] have found that a higher academic year is associated with less stigma, which, in turn, can lead to more positive help-seeking attitudes toward mental health problems. In addition to the university year, these differences might also be related to the age difference between the two years. Previous studies have found an association between lower stigma [10], as well as higher psychological openness, and older age [7].

Psychology students exhibited fewer stigmatizing beliefs about mental illness and more positive attitudes toward help-seeking (psychological openness, propensity to seek help, and indifference to stigma). Several studies [30,31] claim that lower stigma in Psychology students may be explained by psychopathology, based on a biopsychosocial model that allows a more holistic view of the person, rather than an emphasis on diagnosis, which is associated with less negative attitudes toward mental illness and treatment. In short, the study of Psychology can play the role of a facilitator for seeking help, due to its potential to reduce stigma and help in identifying problems [7].

In our sample, some students revealed currently having psychological support, mainly for anxiety and depression symptoms. As expected, these students showed higher levels of psychological openness and propensity to seek help, as well as less stigmatizing beliefs about mental illness (i.e., dangerousness). This can be related to greater empathy, as these types of students are more aware of the difficulties that people with mental health issues go through [29]. Furthermore, participants who claimed to know someone with mental illness were less likely to stigmatize this condition [11,32], suggesting that direct and intimate contact with this group tends to lead to less negative beliefs by minimizing negative emotions [33]. In this sense, Nohr et al. [13] concluded that greater familiarity with mental illness predicts a decrease in label avoidance and negative help-seeking attitudes. A significant percentage of students (45.4%) felt the need to resort to psychological support during or after the COVID-19 pandemic, which is consistent with the literature that identified an increase in psychological issues during this time among students, mainly anxiety and depression symptoms [14,15]. However, an increase in the demand for help from mental health services did not accompany this increase in psychological issues [34]. Regarding the change in opinions and attitudes toward mental illness since the COVID-19 outbreak, more than half of the participants indicated that they felt a change toward people with mental illness (i.e., higher tolerance, understanding, or support). This confirms that the pandemic might have had an impact on people’s perspectives of mental health, affecting certain beliefs like the course and duration of the disease, the perceived possibility of treatment, and the susceptibility of personal control, as proposed by O’Connor [16].

Finally, in the cluster analysis, university course proved to be the variable with the highest predictive importance for the clusters’ formation, meaning that the type of course significantly influenced the students’ levels of stigma and help-seeking attitudes. These findings are relevant, as they highlight the importance of mental health education among students of different courses, for reducing stigma, facilitating problem recognition and, consequently, facilitating the search for professional help in universities. Following the type of course, gender stands out as the variable with the greatest predictive importance. As the profile mostly composed of male students (Prejudiced Students) was the one exhibiting higher stigma levels and lower help-seeking attitudes, the importance of investing in male-targeted mental health education should be emphasized [23], as well as stigma-reduction interventions focused on eliminating stereotypes related to traditional gender roles. Moreover, it is suggested that educational initiatives aimed at increasing men’s use of mental health services should focus primarily on psychological openness rather than on stigma-related attitudes or intentions to seek help.

Additionally, contact with mental illness proves to be a great predictor of less stigma and more positive help-seeking attitudes, as most students who reported having a family member with a mental illness or having sought psychological help were grouped in Cluster 1. Given the greater predictive importance of Psychological Openness and Dangerousness, compared to other IBMI and IATSMHS factors, it appears that, as expected, the participants included in the Self-Aware Students cluster had higher levels of Psychological Openness, and those in the Prejudiced Students cluster had higher levels of Dangerousness beliefs.

The investigation poses some limitations, one of them pertaining to the excessive homogeneity regarding gender distribution, given that most of the sample was composed of female students. Additionally, the results cannot be generalized since the sample was not collected at random, and they are based on self-reported measures which carry the risk of social desirability influence. Finally, although the literature on mental illness stigma is vast, the ones that study the relationship between stigma and formal help-seeking in students may still benefit from further research.

Additionally, the findings from this study provide directions for future research involving groups more vulnerable to mental health problems, particularly LGBTQA+ students, who tend to report a higher prevalence of mental health issues [15]. However, support services for this group are even more scarce, highlighting the need for gender-diverse care [35].

Despite the limitations to this study, we consider that through the cluster analysis method, it was possible to delineate two distinct groups of students, which is relevant as it allows us to trace profiles to outline more uniform intervention groups and, in turn, improve intervention outcomes among this population. In short, this study contributes to the development of new interventions better adapted to the specific needs of each group, making the most of the existing resources.

Moreover, since we could not find any published literature on this topic in Portugal that uses the IBMI and the IATSMHS simultaneously, this study represents a novelty in this sense.

## 5. Conclusions

This study confirms that mental illness is a highly stigmatized condition, and that stigma stands out as one of the major barriers preventing students from seeking psychological help, and is moderated by sociodemographic variables. Thus, universities must convey a clear message that mental health problems are common among college students, and that it is natural and desirable to seek professional help. Additionally, it is crucial to invest in research into and development of effective preventive and supportive strategies to reduce stigma and, consequently, facilitate help-seeking. As evidenced in this study, these strategies should consist of not only mental health education to eliminate false beliefs, but also intergroup contact.

## Figures and Tables

**Table 1:** Sociodemographic characteristics of the sample (*N* = 282; M_age_ = 20.04; SD = 2.48).

Variable	Category	*N* (%)
Gender	Male	69 (24.6%)
	Female	212 (75.4%)
Nationality	Portuguese	259 (91.8%)
	Brazilian	13 (4.6%)
	Other	10 (3.6%)
Course	Sociology	54 (19.1%)
	Psychology	85 (30.1%)
	Fashion Design	80 (28.4%)
	Sports Science	63 (22.3%)
College year	1st year	166 (58.9%)
	3rd year	116 (41.1%)
Psychological support	Yes	56 (20.0%)
	No	224 (80.0%)
Psychological support causes	Anxiety (stress, panic attacks)	21 (7.9%)
	Anxiety and depression	16 (6.0%)
	Other	19 (6.1%)
Family member with mental illness	Yes	89 (32.1%)
	No	188 (67.9%)
Other with mental illness	Yes	196 (70.5%)
	No	82 (29.5%)
Need for psychological support during/after the pandemic	Yes	127 (45.4%)
	No	153 (54.6%)
Change in opinions/attitudes toward mental illness	Yes	160 (57.8%)
	No	117 (42.2%)

**Table 2:** Correlations between IBMI and IATSMHS factors—Pearson’s Coefficient.

	Psychological Openness	Propensity to Seek Help	Indifference to Stigma	IATSMHS Total Score
Incurability	−0.278 **	−0.045	−0.286 **	−0.252 **
Disease Recognition	−0.007	0.172 **	0.139 **	0.132 **
Illness as a Cause of Stigma and Discrimination	−0.379 **	−0.249 **	−0.659 **	−0.563 **
Dangerousness	−0.389 **	−0.180 **	−0.350 **	−0.396 **
IBMI Total Score	−0.398 **	−0.056	−0.391 **	−0.364 **

** *p* < 0.001.

**Table 3:** Two-Step Cluster analysis: Self-Aware Students and Prejudiced Students.

Variable	Self-Aware Students *N* = 146	Prejudiced Students *N* = 94	RPI	Statistic	*p*
Course, *N* (%)			1.00	83.186 (3)	<0.001 ^2^
Sociology	35 (72.9)	13 (27.1)			
Psychology	65 (91.5)	6 (8.5)			
Fashion Design	39 (58.2)	28 (41.8)			
Sports Science	7 (13.0)	47 (87.0)			
Gender, *N* (%)			0.96	71.617 (1)	<0.001 ^2^
Male	7 (12.5)	49 (87.5)			
Female	139 (75.5)	45 (24.5)			
Psychological Openness M (SD)	23.88 (3.96)	19.97 (3.96)	0.69	7.481 (238)	<0.001 ^1^
Dangerousness M (SD)	15.21 (4.43)	19.42 (4.41)	0.64	−7.184 (238)	<0.001 ^1^
Family Member with Mental Illness, *N* (%)			0.58	41.546 (1)	<0.001 ^2^
Yes	73 (89.9)	9 (11.0)			
No	73 (46.2)	85 (53.8)			
College Year, *N* (%)			0.50	35.353 (1)	<0.001 ^2^
1st year	63 (45.0)	77 (55.0)			
3rd year	83 (83.0)	17 (17.0)			
Propensity to Seek Help M (SD)	26.49 (4.35)	22.94 (4.42)	0.49	6.149 (238)	<0.001 ^1^
Need for Psychological Support,			0.45	31.720 (1)	<0.001 ^2^
*N* (%)					
Yes	90 (79.6)	23 (20.4)			
No	56 (44.1)	71 (55.9)			
Psychological Support,			0.43	30.112 (1)	<0.001 ^2^
*N* (%)					
Yes	48 (94.1)	3 (5.9)			
No	98 (51.9)	91 (48.1)			
Illness as a Cause of Stigma and Discrimination M (SD)	12.69 (3.32)	15.29 (4.26)	0.38	−5.010 (164.089)	<0.001 ^1^
Indifference to Stigma M (SD)	29.38 (3.78)	26.57 (5.23)	0.32	4.495 (154.818)	<0.001 ^1^
Age M (SD)	20.50 (2.93)	19.31 (1.45)	0.20	3.667 (238)	<0.001 ^1^
Incurability M (SD)	27.30 (7.02)	30.49 (6.13)	0.20	−3.606	<0.001 ^1^
Change in Opinion/Attitudes,			0.11	5.893 (1)	0.015 ^2^
*N* (%)					
Yes	96 (67.1)	47 (32.9)			
No	50 (51.5)	47 (48.5)			
Disease Recognition M (SD)	50.73 (4.37)	50.10 (4.77)	0.03	11.063 (238)	0.289 ^1^

Note: RPI: Relative Predictor Importance; ^1^ *t*-Test; ^2^ Chi-Square Test; Statistic: t or χ^2^.

## Data Availability

The datasets generated and/or analyzed during the current study are available from the corresponding author upon reasonable request.

## Supplementary Materials

The following supporting information can be downloaded at: https://www.mdpi.com/article/10.3390/healthcare12242505/s1, Table S1: Descriptive statistics of IBMI and IATSMHS factors. Table S2: Effect of Gender on IBMI and IATSMHS factors—*t*-Test/Mann-Whitney Test. Table S3: Effect of College Year on IBMI and IATSMHS factors—*t*-Test. Table S4: Effect of Course on IBMI factors—One-Way ANOVA. Table S5: Pairwise comparisons (Course and IBMI factors)—LSD posthoc test. Table S6: Effects of Course on IATSMHS factors—One-Way ANOVA. Table S7: Pairwise comparisons (Course and IATSMHS factors)—LSD posthoc test. Table S8: Effect of Psychological Support on IBMI and IATSMHS Factors—*t*-Test. Table S9: Effects of Familiarity (family member with mental illness) on IBMI and IATSMHS factors—*t*-Test.

## References

[1] Link B.G., Stuart H., Gaebel W., Rossler W., Sartorius N. (2017). On revisiting some origins of the stigma concept as it applies to mental illnesses. The Stigma of Mental Illness—End of the Story?.

[2] Link B.G., Phelan J.C. (2001). Conceptualizing stigma. Annu. Rev. Sociol..

[3] Riffel T., Chen S.P. (2020). Stigma in Healthcare? Exploring the Knowledge, Attitudes, and Behavioural Responses of Healthcare Professionals and Students toward Individuals with Mental Illnesses. Psychiatr. Q..

[4] Corrigan P.W., Watson A.C. (2002). The Paradox of Self-Stigma and Mental Illness. Clin. Psychol. Sci. Pract..

[5] Chandrasekara W. (2016). Help seeking attitudes and willingness to seek psychological help: Application of the theory of planed behavior. Int. J. Manag. Account. Econ..

[6] Cage E., Stock M., Sharpington A., Pitman E., Batchelor R. (2020). Barriers to accessing support for mental health issues at university. Stud. High. Educ..

[7] Stewart G., Kamata A., Miles R., Grandoit E., Mandelbaum F., Quinn C., Rabin L. (2019). Predicting mental health help seeking orientations among diverse Undergraduates: An ordinal logistic regression analysis. J. Affect. Disord..

[8] Velasco A., Cruz I.S.S., Billings J., Jimenez M., Rowe S. (2020). What are the barriers, facilitators and interventions targeting help-seeking behaviours for common mental health problems in adolescents? A systematic review. BMC Psychiatry.

[9] Vidourek R.A., Burbage M. (2019). Positive mental health and mental health stigma: A qualitative study assessing student attitudes. Ment. Health Prev..

[10] Hartini N., Fardana N.A., Ariana A.D., Wardana N.D. (2018). Stigma toward people with mental health problems in Indonesia. Psychol. Res. Behav. Manag..

[11] Kosyluk K.A., Conner K.O., Al-Khouja M., Bink A., Buchholz B., Ellefson S., Fokuo K., Goldberg D., Kraus D., Leon A. (2020). Factors predicting help seeking for mental illness among college students. J. Ment. Health.

[12] Oliveira A.M., Machado D., Fonseca J.B., Palha F., Silva Moreira P., Sousa N., Cerqueira J.J., Morgado P. (2020). Stigmatizing Attitudes Toward Patients With Psychiatric Disorders Among Medical Students and Professionals. Front. Psychiatry.

[13] Nohr L., Lorenzo Ruiz A., Sandoval Ferrer J.E., Buhlmann U. (2021). Mental health stigma and professional help-seeking attitudes a comparison between Cuba and Germany. PLoS ONE.

[14] Di Consiglio M., Merola S., Pascucci T., Violani C., Couyoumdjian A. (2021). The Impact of COVID-19 Pandemic on Italian University Students’ Mental Health: Changes across the Waves. Int. J. Environ. Res. Public Health.

[15] Lee J., Jeong H.J., Kim S. (2021). Stress, anxiety, and depression among undergraduate students during the COVID-19 pandemic and their use of mental health services. Innov. High. Educ..

[16] O’Connor C. (2021). Has the COVID-19 Pandemic Affected Lay Beliefs about the Cause and Course of Mental Illness?. Int. J. Environ. Res. Public Health.

[17] Loureiro L., Dias C., Ferreira P. (2006). ICDM—Um inventário de crenças acerca da doença mental. Rev. Investig. Enferm..

[18] Pereira A., Patrício T. (2016). SPSS: Guia Prático de Utilização: Análise de Dados Para Ciências Sociais e Psicologia.

[19] Fonseca A., Silva S., Canavarro M.C. (2017). Psychometric characteristics of the Inventory of Attitudes toward Seeking Mental Health Services: A study of women during the perinatal period. Psychologica.

[20] Mackenzie C.S., Knox V.J., Gekoski W.L., Macaulay H.L. (2004). An Adaptation and Extension of the Attitudes Toward Seeking Professional Psychological Help Scale. J. Appl. Soc. Psychol..

[21] Bacher J., Wenzig K., Vogler M. (2004). SPSS two-step cluster—A first evaluation. Univ. Erlangennürnb.

[22] Fekih-Romdhane F., Chebbi O., Sassi H., Cheour M. (2021). Knowledge, attitude and behaviours toward mental illness and help-seeking in a large nonclinical Tunisian student sample. Early Interv. Psychiatry.

[23] DeBate R.D., Gatto A., Rafal G. (2018). The effects of stigma on determinants of mental health help-seeking behaviors among male college students: An application of the information-motivation-behavioral skills model. Am. J. Men’s Health.

[24] Sum M., Chan S., Tsui H., Wong G. (2023). Stigma towards mental illness, resilience, and help-seeking behaviours in undergraduate students in Hong Kong. Early Interv. Psychiatry.

[25] Speerforck S., Schomerus G., Pruess S., Angermeyer M.C. (2014). Different biogenetic causal explanations and attitudes towards persons with major depression, schizophrenia and, alcohol dependence: Is the concept of a chemical imbalance beneficial?. J. Affect. Disord..

[26] Baez S., Flichtentrei D., Prats M., Mastandueno R., García A.M., Cetkovich M., Ibáñez A. (2017). Men, women…who cares? A population-based study on sex differences and gender roles in empathy and moral cognition. PLoS ONE.

[27] Rafal G., Gatto A., DeBate R. (2018). Mental health literacy, stigma, and help-seeking behaviors among male college students. J. Am. Coll. Health.

[28] Liddon L., Kingerlee R., Barry J.A. (2018). Gender differences in preferences for psychological treatment, coping strategies, and triggers to help-seeking. Br. J. Clin. Psychol..

[29] Fernandes J.B., Família C., Castro C., Simões A. (2022). Stigma towards People with Mental Illness among Portuguese Nursing Students. J. Pers. Med..

[30] McNealy K.R., Lombardero A. (2019). Somatic presentation of mental health concerns, stigma, and mental health treatment engagement among college students. J. Am. Coll. Health.

[31] Masedo A., Grandó P., Saldivia S., Vielma-Aguilera A., Castro-Alzate E.S., Bustos C., Romero-López-Alberca C., Pena-Andreu J.M., Xavier M., Moreno-Küstner B. (2021). A multicentric study on stigma towards people with mental illness in health sciences students. BMC Med. Educ..

[32] Lyndon A.E., Crowe A., Wuensch K.L., McCammon S.L., Davis K.B. (2019). College students’ stigmatization of people with mental illness: Familiarity, implicit person theory, and attribution. J. Men. Health.

[33] Hantzi A., Anagnostopoulos F., Alexiou E. (2019). Attitudes Towards Seeking Psychological Help: An Integrative Model Based on Contact, Essentialist Beliefs About Mental Illness, and Stigma. J. Clin. Psychol. Med. Settings.

[34] Upton E., Clare P.J., Aiken A., Boland V.C., Torres C.D., Bruno R., Hutchinson D., Kypri K., Mattick R., McBride N. (2021). Changes in mental health and help-seeking among young Australian adults during the COVID-19 pandemic: A prospective cohort study. Psychol. Med..

[35] Jatchavala C., Udomratn P., Bährer-Kohler S., Bolea-Alamanac B. (2019). Women, and Gender, Diversity and Mental Health. Diversity in Global Mental Health.

