# Supplementary material for: Mental Health Awareness: Stigma and Help-Seeking Among Portuguese College Students
Source: Healthcare (Basel). 2024 Dec 11;12(24):2505. doi: 10.3390/healthcare12242505 (PMC11675085; doi:10.3390/healthcare12242505)
Supplement: Supplementary file 1 [file healthcare-12-02505-s001.zip › healthcare-3309058-supplementary.pdf]

Table S1

Statistics Analysis of IBMI and IATSMHS factors

Variable	Min.	Max.	*M (SD)*
Incurability	10	45	28.45 (6.89)
Disease Recognition	30	60	50.42 (4.59)
Illness as a Cause of Stigma and Discrimination	8	30	13.70 (4.02)
Dangerousness	7	29	17.02 (5.01)
IBMI total score	102	191	146.81 (15.43)
Psychological Openness	11	32	22.27 (4.55)
Indifference to Stigma	8	32	28.25 (4.53)
Propensity to Seek Help	6	32	25.04 (4.82)
IATSMHS total score	34	96	75.55 (10.59)

Table S2

Effect of Gender on IBMI and IATSMHS factors – T-Test / Mann-Whitney Test

Variable	Gender	*N*	*M (SD)/MR*	*t (df)/U*	*p*
Incurability	Male	65	29.92 (6.15)	2.014 (270)	.045*
	Female	207	27.96 (7.05)		
Disease Recognition	Male	67	49.52 (5.25)	7811.50	.049*
	Female	201	50.71 (4.33)		
Illness as a Cause of Stigma and Discrimination	Male	65	14.62 (4.25)	5560.00	.023*
	Female	210	13.39 (3.89)		
Dangerousness	Male	66	18.30 (4.93)	2.460 (272)	.015*
	Female	208	16.58 (4.96)		
IBMI Total Score	Male	58	151.00 (15.23)	2.419 (246)	.016*
	Female	190	145.45 (15.30)		
Psychological Openness	Male	68	94.98	10235.50	.001**
	Female	211	154.51		
Propensity to Seek Help	Male	66	107.61	9034.50	.001**
	Female	211	148.82		
Indifference to Stigma	Male	69	126.21	8265.50	.086
	Female	211	145.17		
IATSMHS Total Score	Male	66	97.22	9588.50	.001**
	Female	209	150.88		

Note: MR = Mean Rank

*p<.05; **p<.001

Table S3

Effect of College Year on IBMI and IATSMHS factors – T-Test

Variable	College Year	*N*	*M (SD)*	*t (df)*	*p*
Incurability	1st	163	29.50 (6.27)	3.093 (271)	.002*
	3rd	110	26.91 (7.47)		
Disease Recognition	1st	155	50.61 (4.57)	0.803 (267)	.423
	3rd	114	50.16 (4.62)		
Illness as a Cause of Stigma and Discrimination	1st	164	13.89 (4.14)	0.955 (274)	.340
	3rd	112	13.42 (3.84)		
Dangerousness	1st	163	17.91 (4.91)	3.645 (273)	.001**
	3rd	112	15.72 (4.88)		
IBMI Total Score	1st	144	149.60 (14.06)	3.421 (247)	.001**
	3rd	105	142.97 (16.44)		
Psychological Openness	1st	164	21.74 (4.37)	-2.325 (278)	.021*
	3rd	116	23.02 (4.71)		
Propensity to Seek Help	1st	163	24.87 (4.50)	-0.692 (2176)	.489
	3rd	115	25.28 (5.26)		
Indifference to Stigma	1st	166	28.10 (4.44)	-0.652 (279)	.515
	3rd	115	28.46 (4.66)		
IATSMHS Total Score	1st	162	74.76 (9.99)	-1.477 (274)	.141
	3rd	114	76.67 (11.34)		

*p<.05; **p<.001

Table S4

Effect of Course on IBMI factors– One-Way ANOVA

Variable	Course	*N*	*M*	*SD*	*F (df1;df2)*	*p*
Incurability	Sociology	51	29.63	6.13	8.459 (3;269)	.001*
	Psychology	84	25.59	6.63		
	Fashion Design	76	30.58	6.84		
	Sports Science	62	28.77	6.72		
Disease Recognition	Sociology	52	50.52	4.48	0.850 (3;269)	.467
	Psychology	79	50.75	3.95		
	Fashion Design	77	50.66	4.85		
	Sports Science	61	49.61	5.10		
Illness as a Cause of Stigma and Discrimination	Sociology	53	13.75	3.92	2.489 (3;272)	.061
	Psychology	85	12.86	3.65		
	Fashion Design	76	13.82	3.88		
	Sports Science	62	14.66	4.57		
Dangerousness	Sociology	53	16.55	4.74	10.347 (3;271)	.001*
	Psychology	83	14.89	4.46		
	Fashion Design	77	18.10	4.80		
	Sports Science	62	18.94	5.15		
IBMI Total Score	Sociology	48	146.50	14.16	5.807 (3;245)	.001*
	Psychology	75	141.20	14.52		
	Fashion Design	70	150.84	15.81		
	Sports Science	56	149.54	15.28		

*p<.001

Table S5

Pairwise comparisons (Course and IBMI factors) – LSD posthoc test

Variable	Course (I)	Course (J)	Mean Difference I-J	*p*
Incurability	Sociology	Psychology	4.04412*	.004*
		Fashion Design	-.95150	.965
		Sports Science	.85326	.984
	Psychology	Fashion Design	-4.99561*	.001**
		Sports Science	-3.19086*	.026*
	Fashion Design	Sports Science	1.80475	.511
Dangerousness	Sociology	Psychology	1.65560	.262
		Fashion Design	-1.55673	.347
		Sports Science	-2.38831*	.046*
	Psychology	Fashion Design	-3.21233*	.001**
		Sports Science	-4.04392*	.001**
	Fashion Design	Sports Science	.81366	.890
IBMI Total Score	Sociology	Psychology	5.3000	.297
		Fashion Design	-4.34286	.547
		Sports Science	-3.03571	.887
	Psychology	Fashion Design	-9.64286*	.001**
		Sports Science	-8.33571*	.011*
	Fashion Design	Sports Science	1.30714	.997

**p*<.05; ***p*<.001

Table S6

Effects of Course on IATSMHS factors– One-Way ANOVA

Variable	Course	*N*	*M*	*SD*	F(df1;df2)	*p*
Psychological Openness	Sociology	53	22.57	4.07	10.952 (3;279)	.001*
	Psychology	84	24.29	4.28		
	Fashion Design	80	21.39	4.43		
	Sports Science	63	20.46	4.45		
Propensity to Seek Help	Sociology	53	25.06	4.64	10.375 (3;277)	.001*
	Psychology	84	27.23	3.92		
	Fashion Design	79	24.01	5.20		
	Sports Science	62	23.37	4.58		
Indifference to Stigma	Sociology	54	28.24	4.49	6.495 (3;280)	.001*
	Psychology	84	29.88	3.09		
	Fashion Design	80	27.65	4.76		
	Sports Science	63	26.84	5.25		
IATSMHS Total Score	Sociology	53	75.79	8.41	16.274 (3;275)	.001*
	Psychology	82	81.35	8.75		
	Fashion Design	79	73.20	10.76		
	Sports Science	62	70.65	10.88		

**p*<.001

Table S7

Pairwise comparisons (Course and IATSMHS factors) – LSD posthoc test

Variable	Course (I)	Course (J)	Mean Difference I-J	*p*
Psychological Openness	Sociology	Psychology	-1.71968	.137
		Fashion Design	1.17854	.551
		Sports Science	2.10572	.056
	Psychology	Fashion Design	2.89821	.001**
		Sports Science	3.82540	.001**
	Fashion Design	Sports Science	.92718	.746
Propensity to Seek Help	Sociology	Psychology	-2.16959	.185
		Fashion Design	1.04395	.742
		Sports Science	1.68564	.269
	Psychology	Fashion Design	3.21353	.001**
		Sports Science	3.85522	.001**
	Fashion Design	Sports Science	.64169	.958
Indifference to Stigma	Sociology	Psychology	-1.54021	.185
		Fashion Design	.59074	.971
		Sports Science	1.39947	.423
	Psychology	Fashion Design	2.23095	.008*
		Sports Science	3.03968	.001**
	Fashion Design	Sports Science	.80873	.856
IATSMHS Total Score	Sociology	Psychology	-5.56121	.009*
		Fashion Design	2.58992	.590
		Sports Science	5.14729	.032*
	Psychology	Fashion Design	8.15113	.001**
		Sports Science	10.70850	.001**
	Fashion Design	Sports Science	2.55737	.552

$^{*}p<0.05$; $^{**}p<0.001$

Table S8

Effect of Psychological Support on IBMI and IATSMHS Factors – T-Test

Variable	Psychological Support	*N*	*M (SD)*	*t (df)*	*p*
Incurability	Yes	56	28.13 (6.09)	-.464	.643
	No	215	28.60 (7.08)	(269)	
Disease Recognition	Yes	54	49.83 (4.60)	-1.097	.274
	No	213	50.60 (4.59)	(265)	
Illness as a Cause of Stigma and Discrimination	Yes	56	13.77 (3.91)	.087 (272)	.931
	No	218	13.72 (4.05)		
Dangerousness	Yes	56	15.11 (4.17)	-3.315	.001*
	No	217	17.55 (5.10)	(271)	
IBMI Total Score	Yes	52	144.33 (14.68)	-1.382	.168
	No	195	147.64 (15.53)	(245)	
Psychological Openness	Yes	55	24.27 (4.28)	3.835	.001*
	No	223	21.72 (4.45)	(276)	
Propensity to Seek Help	Yes	56	27.91 (3.74)	6.135	.001*
	No	220	24.26 (4.79)	(105.705)	
Indifference to Stigma	Yes	56	28.36 (4.69)	.242 (277)	.809
	No	223	28.19 (4.51)		
IATSMHS Total Score	Yes	55	80.55 (9.55)	4.137	.001*
	No	219	74.16 (10.40)	(272)	

*p<.001

Table S9

Effects of Familiarity (family member with mental illness) on IBMI and IATSMHS factors– T-Test

Variable	Family with mental illness	*N*	*M (SD)*	*t (df)*	*p*
Incurability	Yes	88	29.36 (6.19)	1.378 (266)	.169
	No	180	28.13 (7.21)		
Disease Recognition	Yes	85	51.46 (4.09)	2.474 (262)	.014*
	No	179	49.98 (4.75)		
Illness as a Cause of Stigma and Discrimination	Yes	87	14.03 (3.88)	0.916 (269)	.360
	No	184	13.55 (4.10)		
Dangerousness	Yes	88	16.60 (4.99)	-1.022 (268)	.308
	No	182	17.27 (5.05)		
IBMI Total Score	Yes	83	149.00 (14.10)	1.505 (242)	.134
	No	161	145.86 (16.07)		
Psychological Openness	Yes	88	22.45 (4.24)	0.514 (273)	.608
	No	187	22.16 (4.63)		
Propensity to Seek Help	Yes	88	26.20 (4.53)	2.873 (271)	.004*
	No	185	24.43 (4.87)		
Indifference to Stigma	Yes	89	28.07 (4.90)	-0.489 (274)	.625
	No	187	28.35 (4.36)		
IATSMHS Total Score	Yes	87	76.86 (9.59)	1.470 (269)	.143
	No	184	74.86 (10.87)		

*$p<.05$
